# Respiratory Symptoms and Diminished Lung Functions Associated with Occupational Dust Exposure Among Iron Ore Mine Workers in Iran

**DOI:** 10.2174/1874306402014010001

**Published:** 2020-01-23

**Authors:** Abdollah Gholami, Reza Tajik, Khaula Atif, Amin Allah Zarei, Sedigheh Abbaspour, Gholamheidar Teimori-Boghsani, Mohsen Attar

**Affiliations:** 1 Social Determinants of Health Research Center, Faculty of Health, Department of Occupational Health Engineering, Birjand University of Medical Sciences, Birjand, Iran; 2 Department of Occupational Health Engineering, School of Public Health, Arak University of Medical Sciences, Arak, Iran; 3 Health Care Administrator, National University of Medical Sciences, Rawalpindi, Pakistan; 4 Department of Environmental Health Engineering, School of Health, Torbat Heydariyeh University of Medical Sciences, Torbat Heydariyeh, Iran; 5 Health Sciences Research Center, Torbat Heydariyeh University of Medical Sciences, Torbat Heydariyeh, Iran; 6 Department of Nursing, School of Nursing and Midwifery, Torbat Heydariyeh University of Medical Sciences, Torbat Heydariyeh, Iran; 7 Vice Chancellery of Health, Torbat Heydariyeh University of Medical Sciences, Torbat Heydariyeh, Iran

**Keywords:** Respiratory symptoms, Lung Function, Occupational Exposure, Iron-Ore Mine, PFT, FVC, FEV1

## Abstract

**Background:**

Dust exposure at quarry mines is inevitable and can result in poor air quality. This research aimed to assess pulmonary symptoms and lung functions of dust-exposed workers at an iron-ore mine in eastern Iran.

**Methods:**

An environmental cross-sectional study sampled 174 dust-exposed mine workers and 93 unexposed administrative employees as the reference group. A standardized questionnaire on respiratory symptoms was completed in accordance with recommendations of the American Thoracic Society(ATS). Calibrated spirometer measured Pulmonary Function Tests (PFTs). Data were analyzed *via* SPSS-21, integrating independent samples t-test, Chi-square and linear or logistic-regression models.

**Results:**

There was no significant variation between dust-exposed and reference groups in terms of age, weight, height, work experience and the number of smokers (*P*>0.05). Mean levels of exposure to inhalable and respirable mineral-dust were 15.09±2.34 and 3.45±2.57 mg/m^3^ respectively. Pulmonary capacities of dust-exposed group were considerably decreased as compared to others (Forced Vital Capacity [FVC] 86.55±13.77 *vs*. 105.05±21.5; Forced Expiratory Volume in 1 second [FEV1] 88.06±16.8 *vs*. 105.81±21.55; FEV1/FVC 103.03±18.17 *vs*. 93.3±12.49; and Peak Expiratory Flow [PEF] 89.82±22.58 *vs*. 98.09±20.60) (*P*<0.001); with a higher prevalence of cough (*P*=0.041), wheezing (*P*=0.032), and dyspnea (*P*=0.035) among formers. Age along with exposure to respirable-dust significantly reduced FVC, FEV1 and FEV1/FVC. Cigarette consumption attenuated FVC and FEV1 on an average of 5 to 9 units.

**Conclusion:**

Controlled occupational dust-exposure is a definitive pre-requisite to reduce respiratory problems among quarry workers, with an explicit consideration towards mineral- mine workers. Modifiable accomplices like smoking and non-compliance of PPEs usage should be amicably resolved.

## 1 INTRODUCTION

In Iran, expansion of iron ore mining is underway to meet the needs of a flourishing construction industry driven by the growing population and escalation of urbanization ensuing upturn in a number of quarry workers [1]. The Iranian government is encouraging mineral exploration to discover future deposits and is supporting plans towards the expansion of existing iron-ore mines [2]. Hence, there is a need for an increased workforce in mining, however, this occupation has inherent safety and health risks. Extraction of iron ore products during mining involves several processes that can produce dust crushing, screening, loading and transport of large quantities of rock. Therefore, mining environments expose workers over time to high concentrations of inhalable mineral dust that can potentially jeopardize their health [3].

Since the recent increase in mining, current estimates are unavailable for the number of mine workers in Iran. However, past estimates claim that in Iran, more than 8000 workers are exposed to mineral and silica dust [1]; while in the United States, 1.7 million are exposed to crystalline silica [4, 5]. The American Conference of Governmental Industrial Hygienists (ACGIH) and the Occupational Health Committee of Iran have both declared 10 mg/m^3^ and 3 mg/m^3^ as threshold limits for an 8-hour workday exposure to total dust and respirable dust, respectively [6]. However, growing concern for the occupational health of workers in the United States is causing a disputing among these existing levels for more conservative guidelines. In this study conducted, Naghizadeh *et al*. measured the exposure range of total dust and crystalline silica levels in iron ore open-pit mines of eastern Iran as 8.28-800.00 mg/m^3^ and 0.012-26.11 mg/m^3^ respectively; whereas the respirable dust and silica were reportedly between 5.26-66.14 mg/m^3^ and 0.01-1.48 mg/m^3^, respectively [7].

Constituents of dust generated by iron ore mining in Iran can vary based on the deposit. Currently, no cohesive literature is available encompassing asbestos contamination in mineral quarries of Iran, nevertheless, the presence of sulfur along with iron in the rock has been reported [8-10]. Various mines contain varied levels of silica-dust as well [11-13], instigating occupational complications like silicosis, bronchitis, renal and pulmonary lesions, oxidative stress and DNA damage [11, 13, 14]. Exposure may even cause tuberculosis, in the absence of silicosis [15, 16]. Such health hazards are often proportionate with the concentration of silica dust, percentage of crystalline silica in the dust, and duration of exposure (acute versus chronic). There are substantial public health concerns shared by the developing and developed nations alike in regard to mine workers' health [17]. The literature validates aggravating respiratory symptoms and attenuating pulmonary functions among mineral-dust exposed workers [18, 19]; including data pertaining to workers exposed to cement dust [20], and results from analyzing mine personnel with long-term exposure to mineral dust [11, 21]. Developing countries, especially Iran, lack formal data about the subject in question and safety of workers [13]. Mineral miners in Iran are non-compliant and fail to follow minimal personal protective measures including using their Personal Protective Equipment (PPE) There are grim and obstinate inadequacies in awareness and education regarding health and safety measures [22-24].

There remains a paucity of data about the respiratory health effects from exposure to iron ore dust generated by mining. The aim of this novel study was to examine respiratory abnormalities and impairments in lung functioning associated with exposure to dust from iron ore mining in Iran. The follow-ing research question was posed: *“Is there a significant association between exposure to iron-ore dust and increased self-reported respiratory symptoms and decreases in pulmonary functioning?"*

## 2 MATERIALS AND METHODS

### 2.1 Subjects Studied

This environmental cross-sectional study was conducted among male workers of an iron-ore quarry located in eastern Iran, as there were no female staff there. It incorporated 267 participants; 174 being dust-exposed mine workers and 93 matched administrative operators as the reference group, both groups had relatable demographic statuses (Table **1**). The research centered on the Declaration of Helsinki and its amendment; while written ethical informed consent was received from all eligible participants [25].

### 2.2 Protocol

In this study, two inventories were assimilated; first self-made with personal information (age, height, weight, work experience, smoking, use/ non-use of masks), and second standardized questionnaire suggested by American Thoracic Society (ATS) [26]. The latter evaluated pulmonary symptoms (chronic cough, wheezing, shortness of breath, mucus and/or bronchitis), nose and eye signs, smoking, medical and family history, occupation, work experience and previous jobs (especially those with a risk of developing pulmonary diseases).

Inclusion criteria were: one year or more of work experience in mine and lack of respiratory tract illnesses approved by concerned specialists (asthma, sinusitis, and other infections); while participants with any contraindications for spirometry (stroke, recent surgery on eye, chest or abdomen), blood sputum, uncontrolled blood pressure or cold in the last few days were excluded from the study.

Pulmonary Function Tests (PFTs) were measured by portable calibrated spirometers (COMPACT Model, by Vitalograph, England) [27] at the factory site by trained and skilled technicians under due supervision of authors; preceded by passable training of respondents to use the spirometer. Spirometry was adjusted for age, gender, weight, height for each individual participant and was performed according to ATS (American Thoracic Society) standards; participants being in the standing position and wearing nose-clips. Performed thrice, the highest values for Forced Vital Capacity (FVC), Forced Expiratory Volume in the first second (FEV1), the ratio of forced expiratory volume in 1 second to forced vital capacity (FEV1/FVC) and Peak Expiratory Flow (PEF) were selected independently from three curves.

Concentrations of airborne inhalable and respirable mineral-dust fractions at workplace were assessed by NIOSH 0600 method; air samples were taken from different working units about mean 35 min and collected on Mixed Cellulose Membrane (MCE) Filter with 0.8-sized pore, placed in a 25 mm nylon cyclone, connected to SKC sampling pump with 1.7 l/min flow rate, and calibrated by a digital automatic calibrator [28]. To avoid moisture, filters were placed in the desiccator before and after sampling and evaluated by a digital scale with a 0.00001gr precision.

### 2.3 Data Analyses

Demographic variables and PFTs were expressed as Mean ± SD and compared by Independent t-test. Prevalence of respiratory symptoms was presented as percentages and compared *via* the Chi-square test. Single-sample Kolmogrov-Smirinov” test examined mean distribution normality. Prevalence and Odds Ratios (ORs) were estimated for dichotomous variables of respiratory symptoms. The 95% Confidence Interval (CI) of the OR point estimate was used for significance testing. Logistic regression analyses were used to adjust an OR for a priori-selected potential confounders. Final adjusted ORs and 95% CIs were calculated after controlling for confounding variables (age, weight, height, work experience, cigarette smoking and respirable dust). Also, using Multiple linear regression analysis, the simultaneous effects of confounding variables on pulmonary functions were tested. SPSS-21 (Inc. USA) analyzed data; P<0.05 was considered as significant.

## 3 RESULTS

Out of 267 participants, 174 were mineral-dust-exposed. Table **1** illustrates demographic characteristics. Independent samples t-test did not reveal any significant difference between independent variables of the two groups (P>0.05). Mean exposure levels of inhalable and respirable dust among the exposed group were 15.09±2.34 and 3.45±2.57 mg/m^3^, respectively. Table **2** highlights that all PFT parameters (FVC, FEV1, FEV1/FVC and PEF) were significantly lower among exposed than others (P<0.05).

Table **3** depicts that the frequency of respiratory symptoms was higher among exposed than non-exposed respondents: cough (20.7% *vs*. 10.8%), phlegm (11.5% *vs*. 9.7%), wheezing (11.5% vs. 3.2%) and shortness of breath (21.3% *vs*. 10.8%), *P*-values for cough, wheezing and dyspnea being 0.041, 0.032 and 0.035 respectively.

Associations of independent variables (age, BMI, smoking) and length of exposure to respirable dust with various pulmonary parameters were analyzed by multiple linear regression analysis (Table **4**). Findings after adjusting for vital confounders were as follows; a significant decrease in FVC(*p*<0.001), FEV1(*p*-0.002) and PEF(*p*-0.012) with an increase in dust exposure, with age and working years, a reduction in FVC(*p*-0.027 & 0.002 respectively) and FEV1/FVC (*p*<0.001 & 0.001 respectively), while cigarette consumption attenuated FVC(*p*-0.049), FEV1(p0.049) and PEF(p0.047). BMI did not cast any significant impact on any of the pulmonary parameters.

Table **5** demonstrates associations between exposure to respirable dust and prevalence of respiratory symptoms using Logistic linear regression analysis. Age, BMI, work experience, smoking habits, and exposure to respirable-dust unmasked a substantial connection between respirable-dust exposure and respiratory symptoms (cough, wheezing and shortness of breath). Cigarette consumption aggravated cough (*p*<0.001) and phlegm (*p*<0.001), while wheezing and dyspnea exacerbated with age (*p*-0.016) and respiratory dust, respectively (*p*-0.049).

## 4 DISCUSSION

Mineral dust is a pervasive chemical hazard encountered in mines, which may cultivate respiratory problems after certain levels of exposure. Air-monitoring is imperative in industrial health to appraise workplace contaminants, apply and comply with control-measures and preclude enhanced levels of occupational exposure. In this study, the exposed mine workers and the unexposed reference workers were evaluated for differences in the prevalence of respiratory symptoms and impairments in lung functioning. Results of air testing in the exposed miner’s environments revealed that exposure rate of inhalable and respirable dust was higher than the Occupational Exposure Limits (OEL) recommended by the Technical Committee of Health Professionals of Iran and occupational exposure standard of the ACGIH [6].

Spirometry is an important, easily accessible and cost-effective para-clinical instrument, which detects pulmonary defects; therefore, it is extensively used to screen industrial lung diseases. This study inferred that pulmonary functions were lower among the exposed mine workers than their unexposed reference counterparts group. Literature validates that occupational-dust exposure impedes lung functions and capacities [29-32], as pronounced by Mahmood *et al*. and Zeleke *et al*. about people exposed to silica-containing dust [33, 34], and Isara *et al*. regarding stoneworkers [19]. This data also publicized that FVC, FEV1 and PEF were significantly reduced among exposed than counter-parts, while the least difference observed in FEV1/FVC. Similar results have been concurred by other authors [33]. FEV1 reduction indicates obstructive impairment, which may also depict dust-induced mechanical stimulation [35]. Contrarily, FVC alone cannot justify a change in lung functions as it may be slightly impaired in healthy subjects or in patients with the minimal obstructive pulmonary disease [36]. According to Gomes *et al*. among casting workers, FEV1, FEV1/FVC, FEF25-75 and PEF were less while FVC and Vital Capacity (VC) did not condense significantly in exposed than others [37].

As there was no significant difference between this study’s participant’s demographic variables, therefore, the reduction in FVC, FEV1 and PEF among dust-exposed mine workers could be attributed to occupational dust workplace, which was further diminished among dust-exposed cigarette smokers. Hochgatterer and *et al*. concurred that duration of dust-exposure and cigarette smoking cast cumulative effects to deteriorate lung functions [12].

Cigarette smoking imparts plummeting and perpetual hazards on the respiratory system, which in combination with dust-inhalation further casts resonating health perils on victims [38]; especially coal-mine workers [39]. Similar inferences were documented from Iran [40], Tanzania [41] and Ethiopia [42]; although researchers from Brazil defied such subtext [43]. Kuempel *et al*. reported that emphysema was more severe amid coal miners than non-miners among ever and never smokers; while cumulative exposure to respirable coal-mine dust was a considerable predictor of emphysema severity once accounted for cigarette smoking, age at death, and race [44].

Symptoms like cough, wheezing and dyspnea significantly differed among the two study groups. This finding is consistent with the results of Kakooei *et al*. [20] and Isara *et al*. [21] encircling cement and quarry workers respectively, which could be attributed to non-usage PPE. In the study of Mamuya *et al*., development workers who had more cumulative exposure to coal dust, had a significantly higher prevalence of acute and chronic respiratory symptoms than other workers [41]. In the study by Hamatui *et al*., exposure to respirable charcoal dust levels was higher than the occupational recommended limit in most units and participants had exposure-related adverse respiratory symptoms such as cough, phlegm and shortness of breath [45]. Ayaaba *et al*,. investigated respiratory disorders among gold miners in Ghana, in which cough was the most observed respiratory symptom [46]. The varying severity of respiratory symptoms in different studies can depend on the type of dust and the amount of exposure. In addition, the use of respiratory PPE along with higher educational levels was a protective factor for silicosis in mine workers [47].

This research recognizes several limitations. Although subject groups were consistent in terms of age, weight, and height, nevertheless, gender-bias and interview bias could not be ruled out. Added investigations like blood tests, chest X-ray and other radiological screenings could yield superior outcomes. None of the respondents provided detailed information about the proper usage of PPE; therefore, its effects on health outcomes of two groups could not be compared. Nonetheless, this study was the first survey of its type from a pertinent area and industry and may provide a way-forward for similar local, national or international surveys. It presented novel first-hand, genuine and useful data; and vetted the posed research question. These findings may lead to the formation of basic strategies to attenuate industrial dust-levels and promote protective measures among mine workers. Smoking should be more vividly discouraged among those already at risk to develop pulmonary disorders due to occupational dust exposure in mining environments.

## CONCLUSION

The hazardous effects of mineral dust on exposed personnel should never be overlooked. Regular assessments of environmental dust in every industry, passable ventilation and adherence to protective measures including availability and use of high-quality PPE must be reinforced at all echelons. Health promotion, occupational exposure assessments and smoking cessation programs are strongly recommended for mine workers.

## Figures and Tables

**Table 1:** Demographic variables of respondents (n=267).

Variable	Exposed Group (n=174)	Control Group (n=93)	P
Age (years), Mean±SD	35.54±8.76	37.30±7.98	t=-1.608, df=265*p*-value= 0.109
Height (cm), Mean±SD	173.41±7.51	172.28±6.39	t=1.235, df=265*p*-value= 0.218
Weight (kg), Mean±SD	74.25±13.74	74.36±11.69	t=-0.064, df=265*p*-value= 0.949
BMI (kg/m^2^), Mean±SD	24.66±4.05	25.07±3.85	t=-0.809, df=265*p*-value= 0.419
Work experience (years), Mean±SD	9.20±5.5	9.58±5.34	t=-0.542, df=265*p*-value= 0.588
Number of smokers, n (%)	24(13.8)	10(10.8)	χ2=0.504, OR=0.753, *p*-value=0.478

**Table 2:** Comparison of pulmonary function tests among dust-exposed and control groups.

Variables	Exposed Group (n=174)	Control Group (n=93)	P
FVC (%)	86.55±13.77	105.05±21.50	t=-8.540, df=265*p*-value= <0.001
FEV_1_ (%)	88.06±16.8	105.81±21.55	t=-7.43, df=265*p*-value= <0.001
FEV_1_/FVC (%)	103.03±18.17	93.3±12.49	t=4.598, df=265*p*-value= <0.001
PEF (%)	89.82±22.58	98.09±20.60	t=-2.940, df=265*p*-value= 0.004

**Table 3:** Relation between respiratory symptoms among the exposed and control groups.

Parameter		Exposed Group	Control Group	*p*-value	cOR*	95% CI
Cough	Yes	36(%20.7)	10(%10.8)	**0.041**	2.165	1.021-4.591
	No	138(%79.3)	83(%89.2)	-	-	-
Phlegm	Yes	20(%11.5)	9(%9.7)	0.649	1.212	0.528-2.781
	No	154(%88.5)	84(%90.3)	-	-	-
Wheezing	Yes	20(%11.5)	3(%3.2)	**0.032**	3.896	1.126-13.477
	No	154(%88.5)	90(%96.8)	-	-	-
Dyspnea	Yes	37(%21.3)	10(%10.8)	**0.035**	2.242	1.059-4.745
	No	137(%78.7)	83(%89.2)	-	-	-

* Crude odds ratio

**Table 4:** Association between confounding variables and lung functions among exposed subjects

**Parameters**	**Variables**	**Regression** **Coefficient (*β*)**	**95% CI**	***p-*value^††^**
**FVC**	Age	0.380	0.045-(0.715)	0.027
	BMI	0.094	-0.395-(0.584)	0.704
	Work experience	-0.875	-1.411-(-0.339)	0.002
	Smoking	-5.579	-11.122-(-0.035)	0.049
	Respirable dust	-1.638	-2.403-(-0.873)	<0.001
**FEV_1_**	Age	-0.391	-0.810-(0.027)	0.067
	BMI	0.165	-0.447-(0.776)	0.596
	Work experience	-0.182	-0.851-(0.487)	0.592
	Smoking	-6.691	-13.883-(0.040)	0.049
	Respirable dust	-1.559	-2.514-(-0.604)	0.002
**FEV_1_/FVC**	Age	-0.976	-1.439-(-0.512)	<0.001
	BMI	-0.187	-0.865-(0.490)	0.586
	Work experience	0.982	0.241-(1.724)	0.01
	Smoking	-1.101	-8.768-(6.567)	0.777
	Respirable dust	-0.209	-1.267-(0.850)	0.698
PEF	Age	-0.047	-0.624-(0.529)	0.872
	BMI	0.728	-0.115-(1.570)	0.09
	Work experience	-1.166	-1.088-(0.756)	0.722
	Smoking	-9.670	-19.206-(-0.135)	0.047
	Respirable dust	-1.699	-3.015-(-0.383)	0.012

^††^Multiple linear regression analysis.

**Table 5:** Relation between confounding variables and respiratory symptoms among the exposed personnel.

**Parameters**	**Variables**	** cOR (95% CI) **	*** p- * value ^ †† ^**	aOR (95% CI) ^ * ^	*** p- * value ^ †† ^**
Cough	Age	1.016 (0.974-1.059)	0.463	1.009 (0.944-1.078)	0.799
	BMI	0.986 (0.899-1.08)	0.755	0.988 (0.895-1.09)	0.805
	Work experience	1.026(0.962-1.093)	0.439	1.030 (0.928-1.143)	0.579
	Smoking	5.25 (2.110-13.06)	<0.001	5.735 (2.244-14.65)	<0.001
	Respirable dust	0.995 (0.862-1.148)	0.943	0.961 (0.819-1.127)	0.624
Phlegm	Age	1.048 (0.996-1.01)	0.72	1.056 (0.969-1.150)	0.216
	BMI	0.967 (0.859-1.088)	0.579	0.973 (0.848-1.116)	0.695
	Work experience	1.056 (0.979-1.139)	0.159	1.034 (0.908-1.177)	0.611
	Smoking	13.256 (4.65-37.81)	<0.001	19.599 (5.882-65.307)	<0.001
	Respirable dust	0.988 (0.822-1.186)	0.896	0.898 (0.712-1.133)	0.366
Wheezing	Age	1.093 (1.035-1.154)	<0.001	1.099 (1.018-1.187)	0.016
	BMI	0.990 (0.882-1.112)	0.871	0.996 (0.879-1.129)	0.951
	Work experience	1.095 (1.017-1.178)	0.016	0.992 (0.889-1.107)	0.890
	Smoking	1.675 (0.508-5.518)	0.396	1.838 (0.504-6.707)	0.357
	Respirable dust	1.050 (0.881-1.252)	0.583	1.055 (0.872-1.276)	0.583
Dyspnea	Age	1.024 (0.983-1.067)	0.256	1.004 (0.939-1.073)	0.915
	BMI	1.017 (0.930-1.112)	0.714	1.037 (0.943-1.141)	0.451
	Work experience	1.049 (0.986-1.117)	0.129	1.047 (0.945-1.160)	0.380
	Smoking	1.647 (0.626-4.331)	0.312	1.689 (0.619-4.606)	0.306
	Respirable dust	1.140 (0.996-1.305)	0.058	1.155 (1.00-1.333)	0.049

^††^Binary logistic regression; *Adjusted odds ratio.
